# MmisAT and MmisP: an efficient and accurate suite of variant analysis toolkit for primary mitochondrial diseases

**DOI:** 10.1186/s40246-023-00557-6

**Published:** 2023-11-27

**Authors:** Shuangshuang Huang, Zhaoyu Wu, Tong Wang, Rui Yu, Zhijian Song, Hao Wang

**Affiliations:** 1 Department of Clinical Laboratory, Children’s Hospital, Zhejiang University School of Medicine, National Clinical Research Center for Child Health, Hangzhou, China; 2 OrigiMed, 5th Floor, Building 3, No.115 Xin Jun Huan Road, Minhang District, Shanghai, China; 3 Department of Clinical Laboratory, The Affiliated Hospital of Guangdong Medical University, Zhanjiang, China; 4 Department of Ophthalmology, Children’s Hospital, Zhejiang University School of Medicine, National Clinical Research Center for Child Health, Hangzhou, China

**Keywords:** Machine learning, Genetics, Variant, Pathogenicity predictor, Primary mitochondrial disease

## Abstract

Recent advances in next-generation sequencing (NGS) technology have greatly accelerated the need for efficient annotation to accurately interpret clinically relevant genetic variants in human diseases. Therefore, it is crucial to develop appropriate analytical tools to improve the interpretation of disease variants. Given the unique genetic characteristics of mitochondria, including haplogroup, heteroplasmy, and maternal inheritance, we developed a suite of variant analysis toolkits specifically designed for primary mitochondrial diseases: the Mitochondrial Missense Variant Annotation Tool (MmisAT) and the Mitochondrial Missense Variant Pathogenicity Predictor (MmisP). MmisAT can handle protein-coding variants from both nuclear DNA and mtDNA and generate 349 annotation types across six categories. It processes 4.78 million variant data in 76 min, making it a valuable resource for clinical and research applications. Additionally, MmisP provides pathogenicity scores to predict the pathogenicity of genetic variations in mitochondrial disease. It has been validated using cross-validation and external datasets and demonstrated higher overall discriminant accuracy with a receiver operating characteristic (ROC) curve area under the curve (AUC) of 0.94, outperforming existing pathogenicity predictors. In conclusion, the MmisAT is an efficient tool that greatly facilitates the process of variant annotation, expanding the scope of variant annotation information. Furthermore, the development of MmisP provides valuable insights into the creation of disease-specific, phenotype-specific, and even gene-specific predictors of pathogenicity, further advancing our understanding of specific fields.

**Supplementary Information:**

The online version contains supplementary material available at 10.1186/s40246-023-00557-6.

## Introduction

Mitochondria, presented in all nucleated cells, generate adenosine triphosphate (ATP) through Oxidative Phosphorylation (OXPHOS) to provide energy for cellular processes. Approximately 1,500 proteins have been found in mitochondria, most of which are transcribed and translated by nuclear genome genes (nDNA). However, 37 genes encoded by the mitochondrial genome (mtDNA) including 13 protein-coding mtDNA essential for the OXPHOS pathway, 22 mt-tRNA, 2 mt-rRNA [1]. Since the identification of pathogenic variants in the mitochondrial genome in 1988 [2] and the subsequent discovery of pathogenic variants in the nuclear genome encoding proteins required for mitochondrial function in 2000–2001 [3–5], over 350 gene causing primary mitochondrial diseases have been identified [6]. The diagnosis of mitochondrial diseases is widely recognized as a complex and challenging task that requires a comprehensive evaluation through biochemical, histochemical, and molecular-level assays. Despite great progress has been made in sensitivity of mitochondrial heteroplasmy detection using next-generation sequencing (NGS) technologies [7] such as whole genome sequencing (WGS), whole exome sequencing (WES) and RNA sequencing (RNA-seq), the interpretation of disease-associated variants from large-scale NGS data remains a challenge for clinicians [8]. Several annotation tools are available for variant interpretation in primary mitochondrial diseases, such as Annovar [9], Variant Effect Predictor (VEP) [10] and snpEff [11]. However, these tools are mostly used for genome-wide annotation but lack specificity for mitochondrial diseases due to the differences between mitochondrial and nuclear DNA. Mitochondrial annotation can be a challenging task due to their unique genetic characteristics, including haplogroup, heterogeneity, and matrilineal inheritance [12]. Fortunately, the complete sequence length of human mitochondrial genome is only about 16.5 kb, and online resources such as HmtDB [13] and HmtVar [14] have almost annotated all possible mtDNA variants. These resources provide clinicians with efficient ways to identify and annotate pathogenic variants in mitochondrial DNA.

Identifying of disease-specific pathogenic variants from a large number of rare variants is another challenge for clinical diagnosis. Although various genome-wide pathogenicity predictors, such as Polyphen-2 [15], SIFT [16], CADD [17], and PROVEAN [18] have been widely used to assess the potential damage of variants, they perform poorly in predicting mitochondrial-genome variants [19]. To overcome this issue, mitochondrial-genome pathogenicity predictors, such as MToolBox [20], APOGEE [21] and Mitoclass [22] have been developed. When analyzing the effects of rRNA variants on mitochondrial genomes, direct methods to determine their pathogenicity are often lacking. In the absence of such an approach, Elson et al. [23] devised an indirect method called Heterologous Inferential Analysis (HIA) that can be used to predict the disruptive potential of a large number of mt-rRNA variants. These predictors exploit desirable features such as structural, conservation of genes, population allele frequencies, evolutionary conservation, tertiary structure, ribosomal RNA (rRNA) variants and biochemical analysis [24], but they also have their limitations. Recent studies have shown that it is possible to develop pathogenicity predictors for rare variants by training specific predictors on variant datasets, disease-specific features[25], specific genes [26], and gene families [27]. These studies yielded promising results that performed well in predicting the pathogenicity of rare variants. For instance, Majithia et al. used a pooled functional assay of human macrophages and supervised machine learning to identify PPARG missense variant known to cause dominant lipid dystrophy and type 2 diabetes [28]. Also, Zhang et al. [27] developed a disease-specific variant pathogenicity predictor called CardioBoost to estimate the pathogenic probability of rare missense variants in hereditary cardiomyopathies and cardiac arrhythmias with AUCs of 0.91 and 0.96, respectively. Even though training specific pathogenicity predictors for each subtype of primary mitochondrial disease are not feasible due to variants scarcity, Zhang et al. [29] recently developed a random forest predictor that estimates the pathogenicity of rare nonsynonymous variants causing abnormal eye phenotypes. Their study showed that the phenotype-specific pathogenicity predictor could significantly improve accuracy, reduce the cost of pathogenic variants identification, and directly identify pathogenic phenotypes of candidate variants, providing opportunities to develop specific pathogenic predictors for primary mitochondrial diseases.

In this study, we developed a suite of variant analysis toolkits specifically designed for primary mitochondrial diseases: Mitochondrial Missense Variant Annotation Tool (MmisAT) and the Mitochondrial Missense Variant Pathogenicity Predictor (MmisP). MmisAT is an annotation tool that rapidly screens missense variants related to mitochondria in the nuclear genome from Variant Call Format (VCF) file and provides comprehensive annotations for variants [30], which facilitates the interpretation of variants associated with primary mitochondrial diseases. MmisP predicts the pathogenicity of rare missense variants in primary mitochondrial diseases using Logistic Regression algorithm and well-curated disease-specific datasets. Our tool outperforms in distinguishing benign variants from pathogenic ones, prioritizing highly disease-associated variants, as well as selecting variants based on stratified clinical outcomes. In conclusion, our study not only provides a practical tool for the study and diagnosis of primary mitochondrial diseases, but also offers an opportunity to discover novel disease-causing genetic variants.

## Materials and methods

A detail description of the data collection, model development, and validation procedures can be found in the Additional file 1. In brief, we developed MmisAT and MmisP to estimate the pathogenicity of rare missense variants that closely associated with primary mitochondrial disease.

MmisAT was primarily designed to annotate 1448 missense variants in mitochondria by filtering out other genes and variants. To obtain authoritative annotation on representative transcripts of variants, we defined the Matched Annotation by NCBI and EMBL-EBI (MANE) transcripts for each gene. We then collected and collated a total of 349 annotations divided into six categories: Basic annotation, Pathogenicity predictor score, Allele frequency, Tissue expression, Amino acid property and Mitochondrial-specific annotation. Although most annotations were from different sources, we extracted 30 new mitochondria-specific annotations from the physiological and functional characteristics of mitochondria. These new annotations helped to improve the interpretation of variants. All annotations could be divided into three categories based on the data type: integer, Boolean, and continuous. The annotations based on the three levels of variants, genes and transcripts, clearly show the impact of variants (Additional file 4: Table S1). Finally, we constructed MmisAT using python code and a Variant Annotation Tool (VAT) based on the hg19 genome build.

To build and validate the MmisP model, we collected data from ClinVar [31], VariSNP [32] and literature resource. The data for all training and testing sets were limited to 321 genes associated with mitochondrial disease and had to be missense variants of these genes. Variants in the training set Vari_Train are derived from ClinVar (prior to October 2019) and VariSNP, which has a total of 3872 variants and a one-to-one ratio of benign to pathogenic variants. Vari_Train ended up involving only 258 genes, because some of the genes did not have variants of the missense type. The Vari_TestUnbalance testing set was derived from ClinVar (From October 2019 to October 2022), which contained 677 benign variants and 281 pathogenic variants. After removing the variants from Vari_TestUnbalance that are missing any of the predictor disease scores, you get Vari_TestBalance, which contains 256 benign variants and 239 pathogenic variants. Vari_TestThreshold is obtained by leaving the variants in Vari_TestUnbalance that have both REVEL and M-CAP pathogenic scores, and it contains 294 benign variants and 277 pathogenic variants. To compare the performance of the predictor in widely studied genes, we obtained the variant set Vari_Test4Gene for four genes (POLG, SLC19A3, PDHA1, ETHE1) through a literature search, which contained 21 benign variants and 23 pathogenic variants. There is no overlap between the testing set and the training set (Additional file 2 and 3).

Our model consists of 115 features to measure pathogenicity. To determine whether mitochondria-related features could improve the model performance, we selected a subset of 85 features for further validation. To increase the generalization ability of the model, we processed all features for missing values and performed normalization. We evaluated six classification algorithms in machine learning and applied nested cross-validation to select the best algorithm. We used an internal fivefold cross-validation loop to optimize the hyperparameters of each candidate classification algorithm. The mean accuracy and standard deviation of Logistic Regression were calculated in an external cross-validation loop (cv = 10). Logistic Regression assumes the data follows a Bernoulli distribution and uses gradient descent to solve the maximum likelihood function for the parameters to achieve binary classification. After selecting of Logistic Regression as the base classifier, we conducted Logistic Regression training for each primary mitochondrial disease using the entire training variant set to construct the MmisP prediction model (see Additional file 1).

In order to comprehensively evaluate the performance of MmisP and other predictors, we introduced 12 evaluation metrics. To assess the applicability of MmisP to Variant Interpretation Guidelines, we used two testing sets (Vari_Test4Gene and Vari_TestThreshold) to compare with other tools at defined gene range and threshold. To explore the experience of using MmisP in a real environment, we also built simulated exomes containing "causative" disease-causing variants. The idea is to search for new disease-causing variants (321 genes) and newly discovered disease-causing variants of genes associated with mitochondrial disease, then process the exome of randomly selected healthy individuals in the 1000 Genome Project (1000G) and finally, place the found variants into the exome. Each simulated exome contained about 400 variants, and there was a "causative" disease-causing variant in each exome.

## Result

### Overview of MmisAT and the impact of MmisP design on its performance

**Fig. 1:**
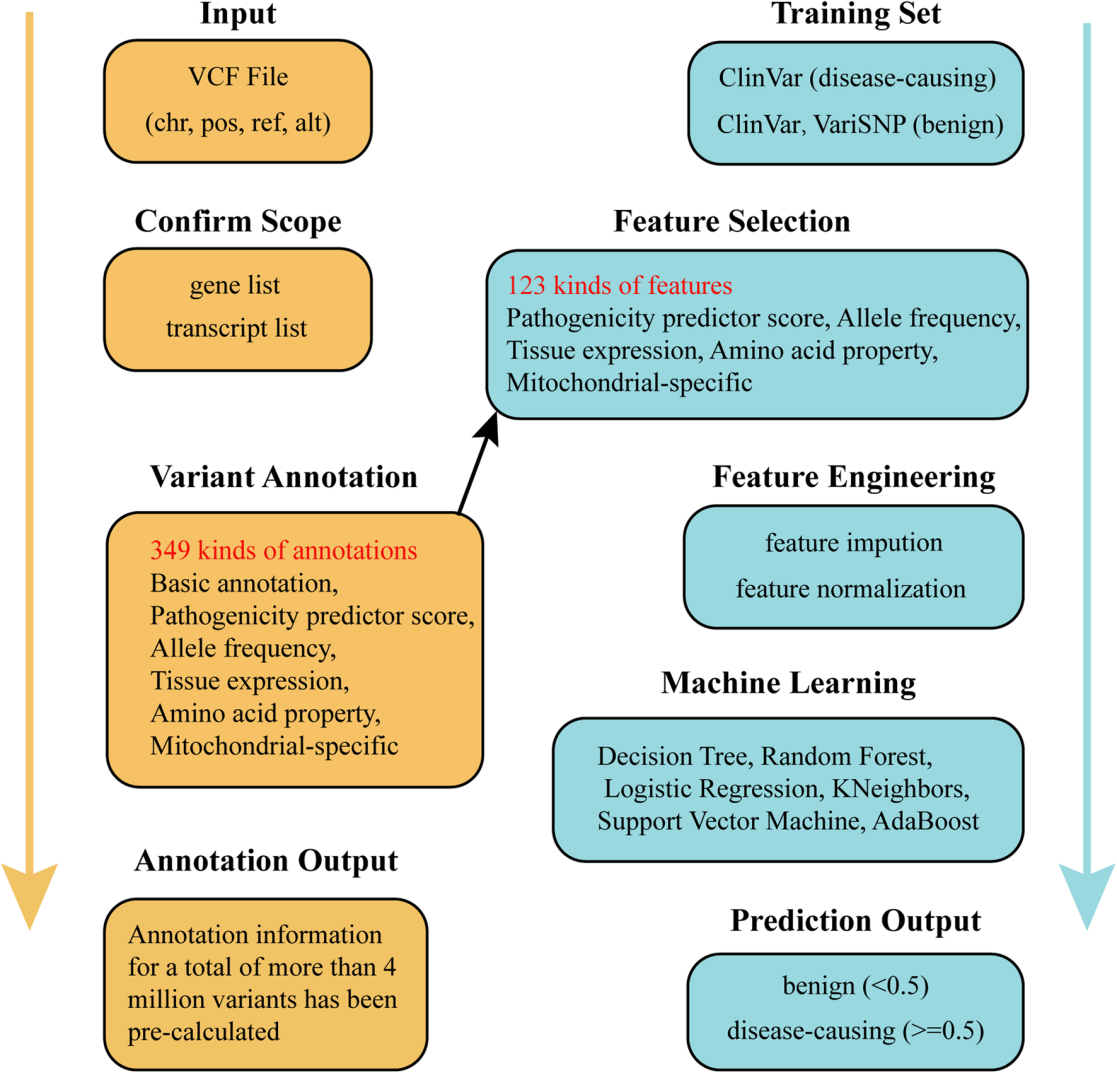
Workflow for MmisAT and MmisP. Left is MmisAT, right is MmisP

MmisAT successfully annotated 13 mtDNA, 321 nDNA with evidence of pathogenicity, and 1127 nDNA whose expression was localized within the mitochondria (Table 1). The corresponding transcript numbers were 13, 1563, and 5366, respectively (Table 1). Notably, MmisP features were selected from the MmisAT annotation (Fig. 1).

**Table 1:** Range of genes and transcripts covered by MmisAT

Total Gene/Transcript	Protein-coding mtDNA	Disease nDNA	Location nDNA
1461	13	321	1127
6942	13	1563	5366

MmisAT can handle protein-coding variants from both nuclear DNA and mtDNA and generate 349 annotation types across six categories (Additional file 4: Table S1). It processes 4.78 million variant data in 76 min, making it a valuable resource for clinical and research applications (Additional file 7: Figure S1). To explore the factors affecting the performance of MmisP, we generated different models and compared their performance on testing datasets. Using Vari_Train, we established a supervised learning classification algorithm to obtain the optimal predictive model for mitochondrial diseases. Although all models performed well with an accuracy rate of over 70% (Table 2), the accuracy metric alone is insufficient to reflect the generalization ability of the model due to the uneven characteristics of Human Whole Exome sequencing data. Therefore, we adopted comprehensive evaluation metrics such as Recall, Precision, F1 Score, Matthew Correlation Coefficient (MCC), and Area Under the Curve (AUC) to assess the performance of each algorithm model. AdaBoost and Logistic Regression showed high Recall rates, both exceeding 80%, while KNeighbors and Decision Tree performed poorly, with recall rates of 71.08% and 68.90%, respectively. Random Forest had the highest accuracy rate of 82.75%, indicating a low probability of misclassifying benign variants as disease-causing variants when using this algorithm. Although Logistic Regression generated the highest F1 score, it was only 0.02% higher than AdaBoost. The MCC values for Logistic Regression, Random Forest and SVM were all greater than 0.7, indicating that these three algorithms could be compared to REVEL [33] and M-CAP [34] at the recommendation threshold of 75% (as detailed below). However, the AUC values for KNeighbors and the Decision Tree were both less than 0.8, which was inconsistent with our expectations for binary models.

**Table 2:** Performance of various algorithmic models

Methods	Accuracy (%)	Precision (%)	AUC	F1Score	Recall (%)	MCC
AdaBoost	80.91	78.84	0.874	0.817	85.01	0.631
Decision Tree	74.30	77.17	0.743	0.727	68.90	0.646
Random Forest	80.96	82.75	0.877	0.802	78.15	0.744
Logistic Regression	81.92	82.01	0.904	0.819	81.92	0.725
KNeighbors	71.25	71.30	0.789	0.710	71.08	0.587
SVM	79.83	81.45	0.880	0.792	77.32	0.711

Vari_Train: 1936 benign, 1936 pathogenic

The features used in our model exhibit different data dimensions and types, and some features may contain redundant information. However, since MmisP focuses on missense variants in mitochondrial diseases, the number of features has little impact on computational time and resource consumption. To evaluate whether features related to mitochondrial function could improve model performance, we constructed six algorithm models using excluded feature subsets (excluding mitochondrial-specific annotations) under the same training conditions. With the exception of Random Forests (which divide nodes by randomly selecting features, so that there may be no significant change in performance for highly linearly correlated features), the performance of all models declines. In particular, accuracy and precision have declined by about 1%, and other metrics have also changed to varying degrees (Table 3). Given the advantage of AUC, we ultimately chose Logistic Regression with a value greater than or equal to 0.9 (0.904, 0.900) to build MmisP. We also use the learning_curve function in the scikit-learn package to evaluate the relationship between MmisP's performance and the size of the training set. As can be seen from Additional file 8: Figure S2, when the training set size is small, the training set error is low, and the cross-validation set error is high. When the size of the training set increases gradually, the model can be generalized better, and the errors of both tend to be stable. MmisP does not underfit or overfit and therefore does not benefit from more training data. In the external tenfold cross-validation loop, the accuracy of MmisP is not only high but also very stable (0.873, 0.827, 0.850, 0.759, 0.770, 0.821, 0.829, 0.790, 0.834, 0.829, mean accuracy: 0.819 ± 0.033), which also shows that the model is universal. In addition, we tested two Logistic Regression models on Vari_TestUnbalance to carefully observe the enhancement effects of disease-specific features on the models.

**Table 3:** Performance of various algorithm models under feature subsets (excluding mitochondrial-specific features)

Methods	Accuracy (%)	Precision (%)	AUC	F1Score	Recall (%)	MCC
Sub_AdaBoost	80.37	77.87	0.871	0.813	85.53	0.654
Sub_Decision Tree	72.39	75.13	0.724	0.705	66.83	0.654
Sub_Random Forest	81.82	83.20	0.880	0.813	79.65	0.739
Sub_Logistic Regression	80.91	81.05	0.900	0.809	80.93	0.702
Sub_KNeighbors	73.71	70.50	0.802	0.756	81.87	0.601
Sub_SVM	79.21	80.47	0.880	0.787	77.32	0.703

Vari_Train: 1936 benign, 1936 pathogenic

**Fig. 2:**
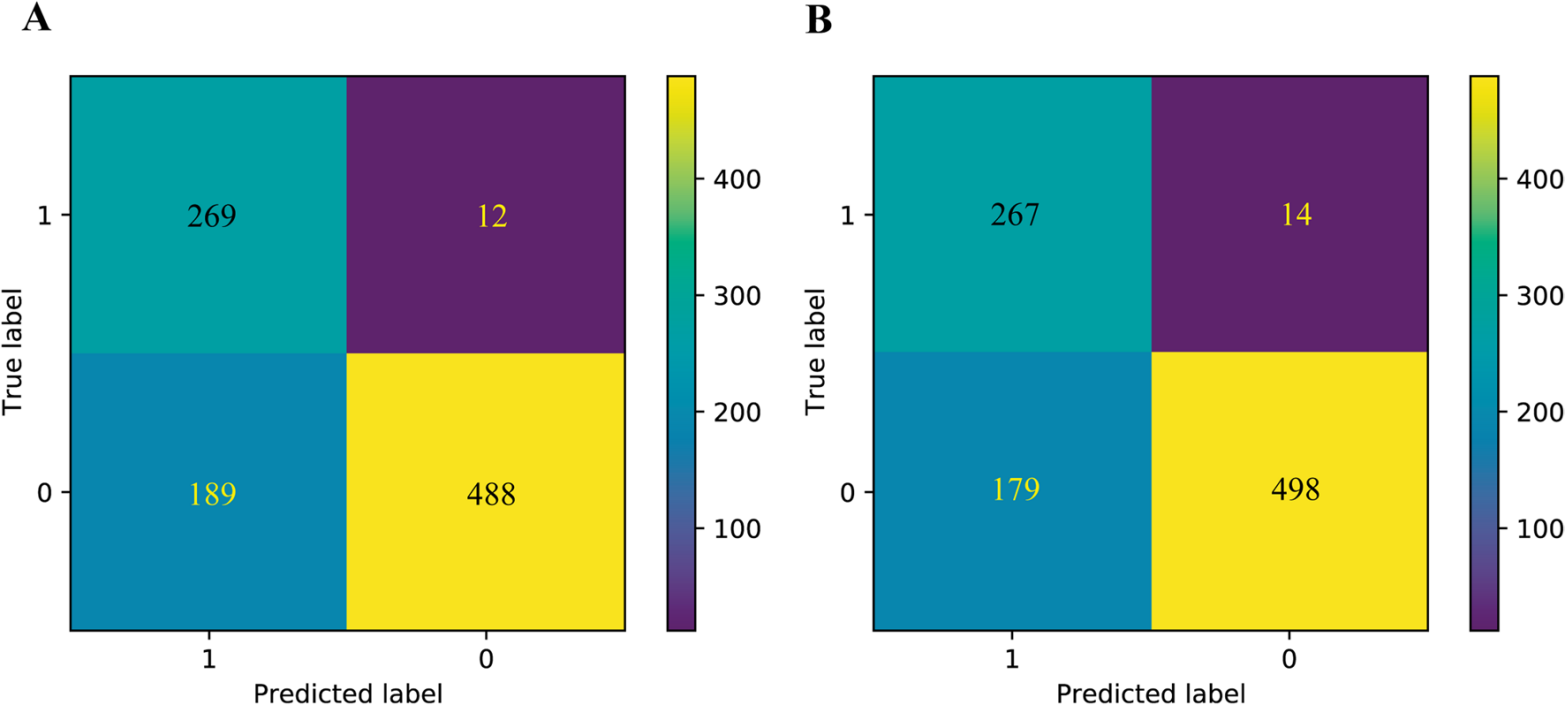
Confusion matrix of Logistic Regression model in two feature backgrounds. **A** A confusion matrix containing 115 complete features. true positive:269, true negative:488, false positive:189, false negative:12. **B** A confusion matrix that does not contain mitochondria-specific features. true positive:267, true negative:498, false positive:179, false negative:14

**Fig. 3:**
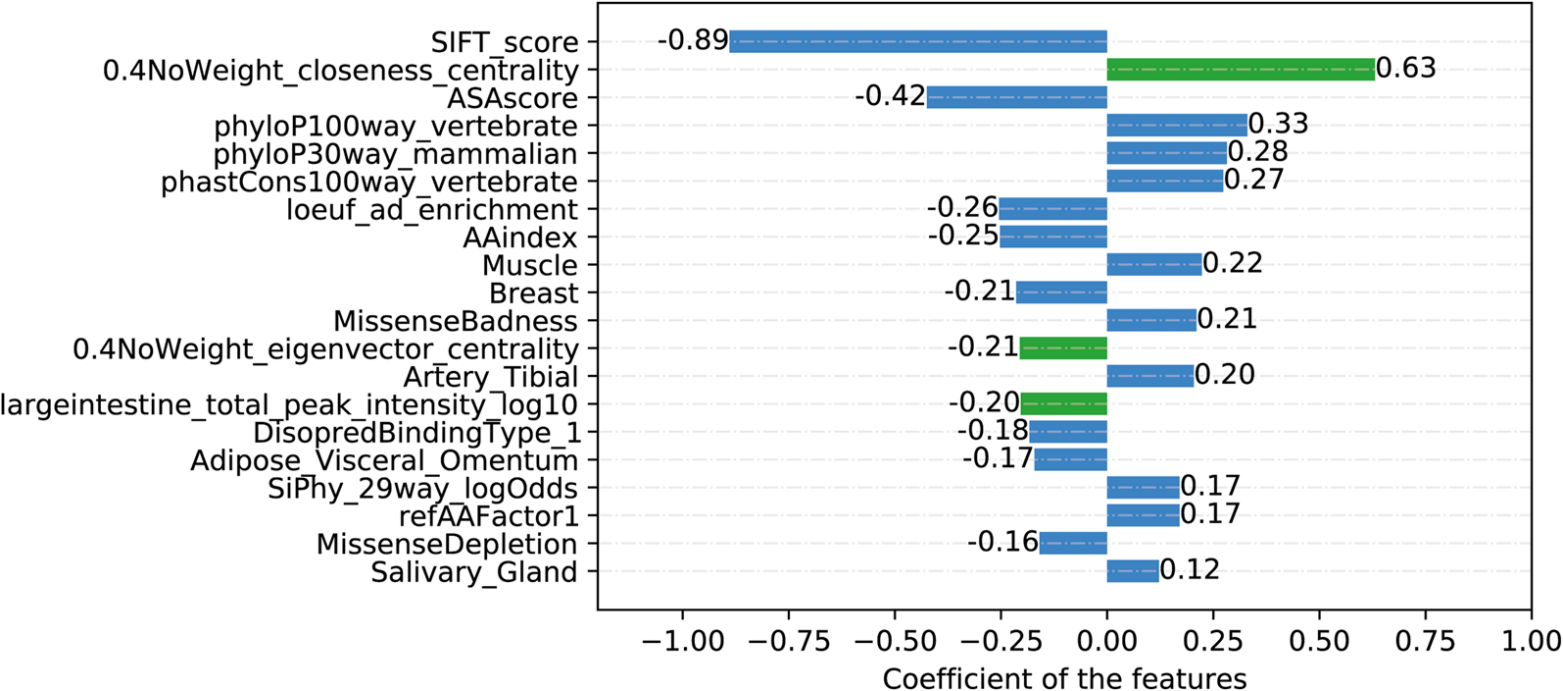
Importance of each feature in the Logistic Regression model. Blue is the common feature and green is the mitochondria-specific feature

The results demonstrated the performance of the predictor, which was evaluated using the confusion matrix shown in Fig. 2. In clinical settings, accurately identifying all pathogenic variants is crucial, which leads to a high true positive (TP) rate (269 > 267). Likewise, it is important to avoid misclassifying true pathogenic variants as benign variants to minimize the false negative (FN) rate (12 < 14). To understand the relative importance of features in the prediction model, we calculated parameter ωi in the Logistic Regression algorithm. Since each feature corresponds to a model parameter ωi, the absolute value of ωi indicates the degree to which it affects the predicted result. Notably, the impact of SIFT_score was the greatest, with a ωi value of 0.89, consistent with our expectations (as shown Fig. 3). Other important features included protein site conservation, population frequency, and tissue expression information of proteins. In addition, the network-centric measures of Closeness_centrality and Eigenvector_centrality ranked second (ωi = 0.63) and twelfth (ωi = 0.21), respectively, highlighting the potential benefit of considering all mitochondrial proteins as an interacting network. Moreover, the peak logarithm of tissue expression (MitoCarta 3.0) ranked fourteenth (ωi = 0.20), indicating the potential of genotype-tissue expression data in improving variant classification accuracy.

### MmisP outperforms genome-wide pathogenicity predictors based on overall classification performance measures

To evaluate the performance of MmisP on Variants of Undetermined Significance (VUS), we compared it to several other genome-wide variant pathogenicity predictors, including M-CAP, REVEL, CADD [35], Eigen [36], and PrimateAI [37], which are renowned for their performance in predicting the pathogenicity of missense variants. We evaluated them using the Vari_TestUnbalance dataset, which emphasizes how the loss of prediction scores limits the utility coverage of pathogenicity predictor. While MmisP and DANN [38] scored all test variants, M-CAP lost nearly 40% missing scores, and the prediction scores of MutationAssessor (8.35%) [39], Polyphen2-HDIV (4.07%) [15], Polyphen2-HVAR (4.07%) [15], Eigen (4.27%) and PrimateAI (4.27%) were also lost. By comprehensively evaluating the performance of all predictors, we found that their Precision (PPV) ranged from 29.47% to 81.74%, and only PrimateAI had a precision exceeding 80% (Table 4). On the other hand, the negative predictive value (NPV) ranges from 78.18 to 100%, with NPVs of more than 90% for all predictors except M-CAP and PrimateAI. The specificity values ranged from 1.03% to 96.76%, and the recall values ranged from 34.94% to 100%.

**Table 4:** Performance of MmisP and other genome-wide tools

Methods	Missing	Precision (%)	NPV (%)	Specificity (%)	FPR (%)	Recall (%)	FNR (%)	Accuracy (%)	MCC	AUC	F1Score
MmisP	0	58.73	97.60	72.08	27.92	95.73	4.27	79.02	0.618	0.938	0.728
MutationAssessor	80	51.20	93.32	63.58	36.42	89.35	10.65	71.30	0.485	0.864	0.651
MutationTaster	1	29.47	100.00	1.03	98.97	100.0	0.00	29.99	0.055	0.760	0.455
PolyPhen2-HDIV	39	48.61	93.29	60.12	39.88	89.71	10.29	68.88	0.457	0.863	0.630
PolyPhen2-HVAR	39	57.00	91.52	73.42	26.58	83.82	16.18	76.50	0.527	0.877	0.679
DANN	0	48.27	93.15	60.27	39.73	89.32	10.68	68.79	0.453	0.815	0.627
Eigen	41	50.71	96.71	63.00	37.00	94.68	5.32	72.08	0.523	0.892	0.660
FATHMM-MKL	0	37.57	95.04	33.97	66.03	95.73	4.27	52.09	0.311	0.793	0.540
M-CAP	383	60.64	89.13	41.69	58.31	94.64	5.36	67.48	0.425	0.854	0.739
MetaLR	10	68.35	90.19	85.07	14.93	77.70	22.30	82.91	0.606	0.901	0.727
MetaSVM	10	69.52	90.68	85.67	14.33	78.78	21.22	83.65	0.623	0.904	0.739
PrimateAI	41	81.74	78.18	96.76	3.24	34.94	65.06	78.63	0.436	0.845	0.490

Vari_TestUnbalance: 677 benign, 281 pathogenic

**Fig. 4:**
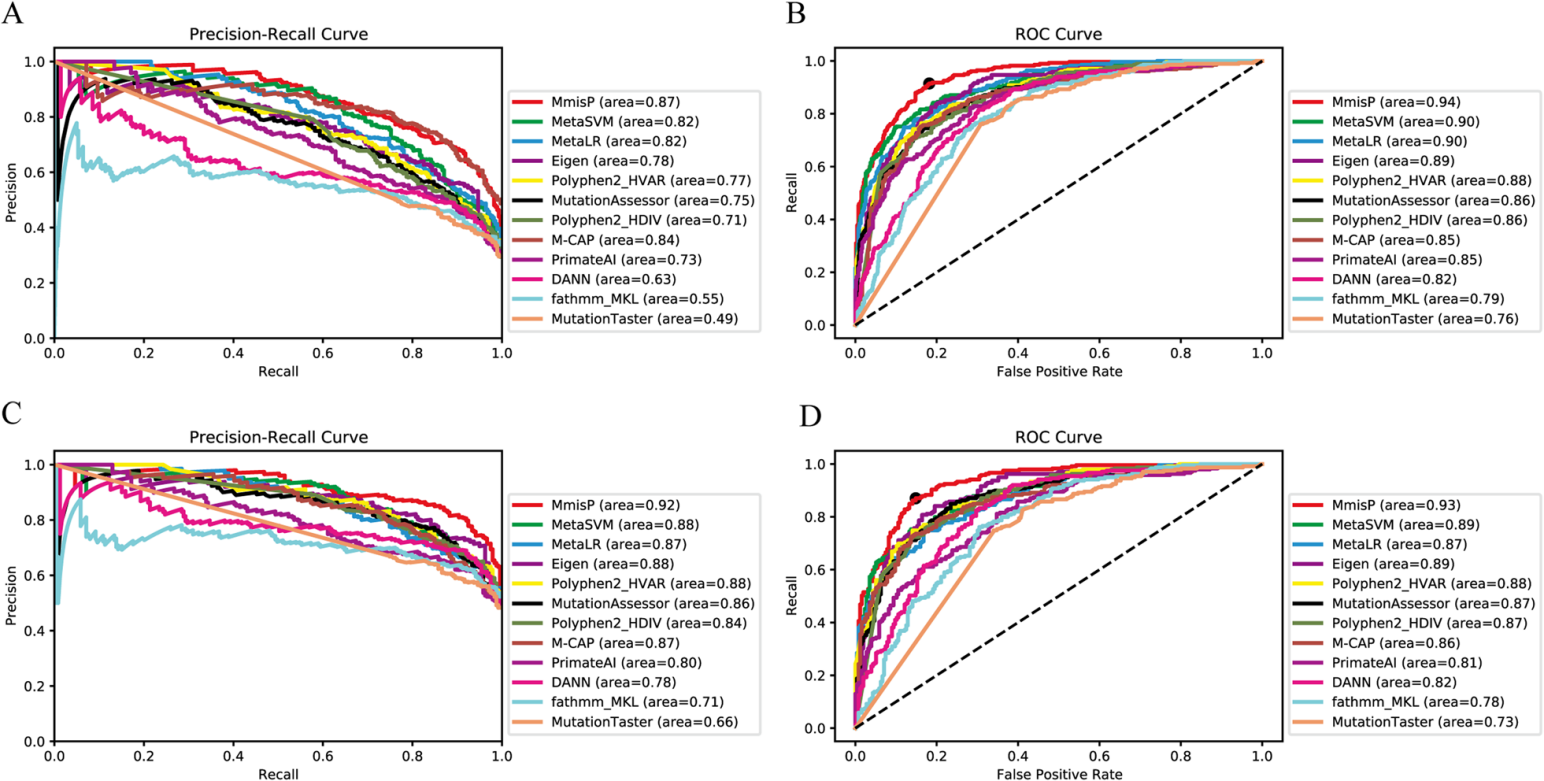
P-R curve and ROC curve of MmisP and other genome-wide pathogenicity predictors under two testing sets. **A** P-R curve under Vari_TestUnbalance. **B** ROC curve under Vari_TestUnbalance. Black dots represent the optimal threshold of MmisP under current circumstances is 0.523. **C** P-R curve under Vari_TestBalance. **D** ROC curve under Vari_TestBalance. Black dots represent the optimal threshold of MmisP under current circumstances is 0.624

Compared to recall, the lower specificity of some pathogenicity predictors suggests that some benign variants may be incorrectly classified as disease-causing. Therefore, it is necessary to establish a more stringent threshold for all pathogenicity predictors. Since the Vari_TestUnbalance dataset is imbalanced, with the number of benign variants exceeding that of disease-causing variants, the Precision-Recall Curve (PRC) is a better indicator of predictor performance (Fig. 4A). Since PRC is sensitive to sample size, we can observe the effect of sample size changes on predictor performance. Among the evaluated predictors, DANN, MutationTaster [40] and fathmm-MKL [41] had average precision of less than 0.7, whereas MmisP among the top with a score of 0.87. The feasibility of MmisP in practical applications has been demonstrated by the unbalanced data of nuclear gene variants associated with mitochondrial diseases. In contrast, the ROC Curve remained unchanged in the case of sample imbalance, so we plotted the ROC Curve and calculated Area Under Curve (AUC). We found that the AUC values of MetaLR (0.901) [42], MetaSVM (0.904) [42] and MmisP (0.938) were greater than 0.9, indicating their advantages r over other predictors (Fig. 4B). For Vari_TestUnbalance, the best classification threshold calculated was 0.624, indicating that MmisP performed better at this threshold. As Vari_TestBalance has a good data balance, the area under the PRC curve for each predictor increased (Fig. 4C), and MmisP still had the largest area. The overall ROC curve did not significantly change (Fig. 4D), with the best classification threshold being 0.523. In summary, MmisP is suitable for application in different variance backgrounds.

### The distribution of disease-causing and benign variants prediction scores

**Fig. 5:**
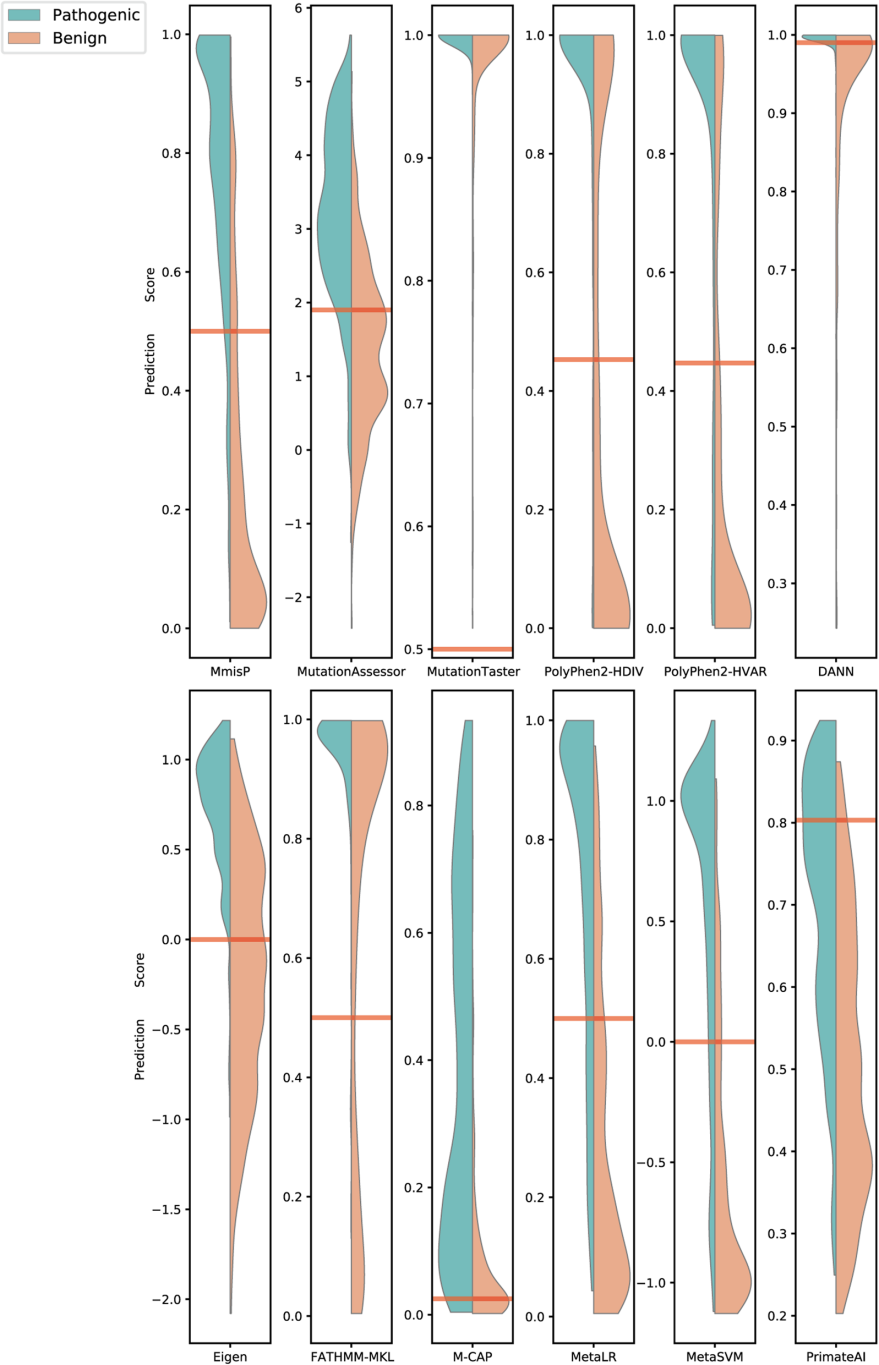
Distribution of prediction scores from MmisP and other genome-wide pathogenicity predictors (Based on the Vari_TestBalance: 256 benign, 239 pathogenic). The red line is the threshold for each tool

To gain deeper insights into the classification process of MmisP and other predictors, we calculated the prediction score for each variant in the Vari_TestBalance dataset and classified them based on a set threshold (0.5 for MmisP). We visualized the distribution of "score" for pathogenic and benign variants using violin plots (Fig. 5). Notably, MutationTaster had the poorest performance with almost all variants receiving prediction scores above the threshold. Eigen, DANN, and fathmm-MKL also performed poorly in classifying benign variants, with around half being falsely classified as disease-causing (Eigen 40.2%, DANN 40.2%, and fathmm-MKL 69.1%). Although M-CAP with high threshold had high sensitivity (the ability to correctly classify variants), it sacrificed specificity, resulting in approximately 57.4% of benign variants being classified as disease-causing. This indicates that an excessive pursuit of high sensitivity may reduce the resolution of exome variant analysis, increase the number of suspicious disease variants, and make it difficult for predictors to identify one or two ‘causative’ variants, hindering the diagnosis of genetic diseases. On the other hand, MmisP, as a proprietary tool, only misclassified 18.7% of benign variants and 9.6% of disease-causing variants. Polyphen2-HVAR also performed well with a misclassification rate of only 26.2% for benign variants and 15.9% for disease-causing variants.

### Performance of MmisP under the ACMG/AMP variant interpretation guidelines

MmisP performed remarkably well in Vari_Test4Gene with a limited range of gene. MmisP's accuracy, F1score, recall and MCC were all higher than the other three tools. PrimateAI's accuracy (100.00%) is perfect, but its recall (45.45%) is the lowest. MmisP's (0.936) AUC value is second only to REVEL's (0.961), which also illustrates its usefulness as a disease-specific pathogenicity predictor (Table 5). We assessed the performance of MmisP using defined thresholds (Probability: Pr > 0.75; Benign variant: Pr < 0.15; Variants of Unknown Significance (VUS): 0.15 ≤ Pr ≤ 0.75) [43], and the PP3/BP4 evidence unique to the Mitochondrial Disease Variants Interpretation Guidelines. We tested MmisP against the guideline-recommended tools REVEL and M-CAP at low classification thresholds using Vari_TestThreshold dataset (Table 6). MmisP had a recall rate of 97.81%, second only to REVEL (98.39%), but significantly better than M-CAP (41.67%, *p* < 0.001). Overall, MmisP correctly classified 56.74% of missense variants, with a slightly higher accuracy than REVEL (52.54%) and M-CAP (52.19%). Additionally, MmisP minimized the number of variants with VUS, with only 38.88% of predicted scores falling into that range, lower than REVEL (45%). M-CAP (34.5%), at the cost of low specificity, only classified a small number of variants as being disease-causing. In conclusion, at the extreme threshold, MmisP can classify 61.12% missense variants as being disease-causing or benign, with 92.84% of them being correctly classified.

**Table 5:** Comparison of MmisP and other predictors in four widely studied genes (POLG, SLC19A3, PDHA1, ETHE1)

Methods	Accuracy (%)	Precision (%)	AUC	F1 Score	Recall (%)	MCC
MmisP	88.63	84.61	0.936	0.897	95.65	0.788
REVEL	79.06	72.41	0.961	0.823	95.45	0.611
PrimateAI	72.09	100.00	0.902	0.625	45.45	0.537
DANN	75.00	82.01	0.900	0.784	86.95	0.507

Vari_Test4Gene: 21 benign, 23 pathogenic

**Table 6:** Performance of MmisP and other tools under recommended thresholds

Methods	MmisP	M-CAP	REVEL
Overall accuracy (%)	56.74	52.19	52.54
The proportion of variants classified with recommended (%)	61.12	65.5	55.0
Accuracy of recommended classifications (%)	92.84	79.68	94.54
The proportion of variants with indeterminate classification (%)	38.88	34.5	45.0
Recall (%)	97.81^*^	41.67	98.39
Precision (%)	89.5	89.28	94.35
Specificity (%)	87.35	97.64	91.34
NPV (%)	97.32	77.99	97.48

Vari_TestThreshold: 294 benign, 277 pathogenic

*p < 0.001

### Performance on simulated disease exomes

In the analysis of Mendelian disease exomes, the major challenge is to identify one or two "causative" disease-causing variants among of hundreds of predicted disease-causing variants, even after applying a standard allele frequency filter to remove common benign variants (MAF > 1%). However, due to the large number of predicted disease-causing variants, it could be difficult to pinpoint the few variants that are truly responsible for the disease, especially with limited resources such as time and cost that make it infeasible to experimentally validate various candidate variants. To address this issue, we randomly selected background exons from 170 and 29 healthy individuals from the 1000 Genomes Project into two groups and introduced a ‘causative’ disease-causing variant into the background exomes to simulate the exomes of Mendelian disorders, which were named Simulated_Exome170 and Simulated_Exome29, respectively (as described in Supplementary Methods).

**Fig. 6:**
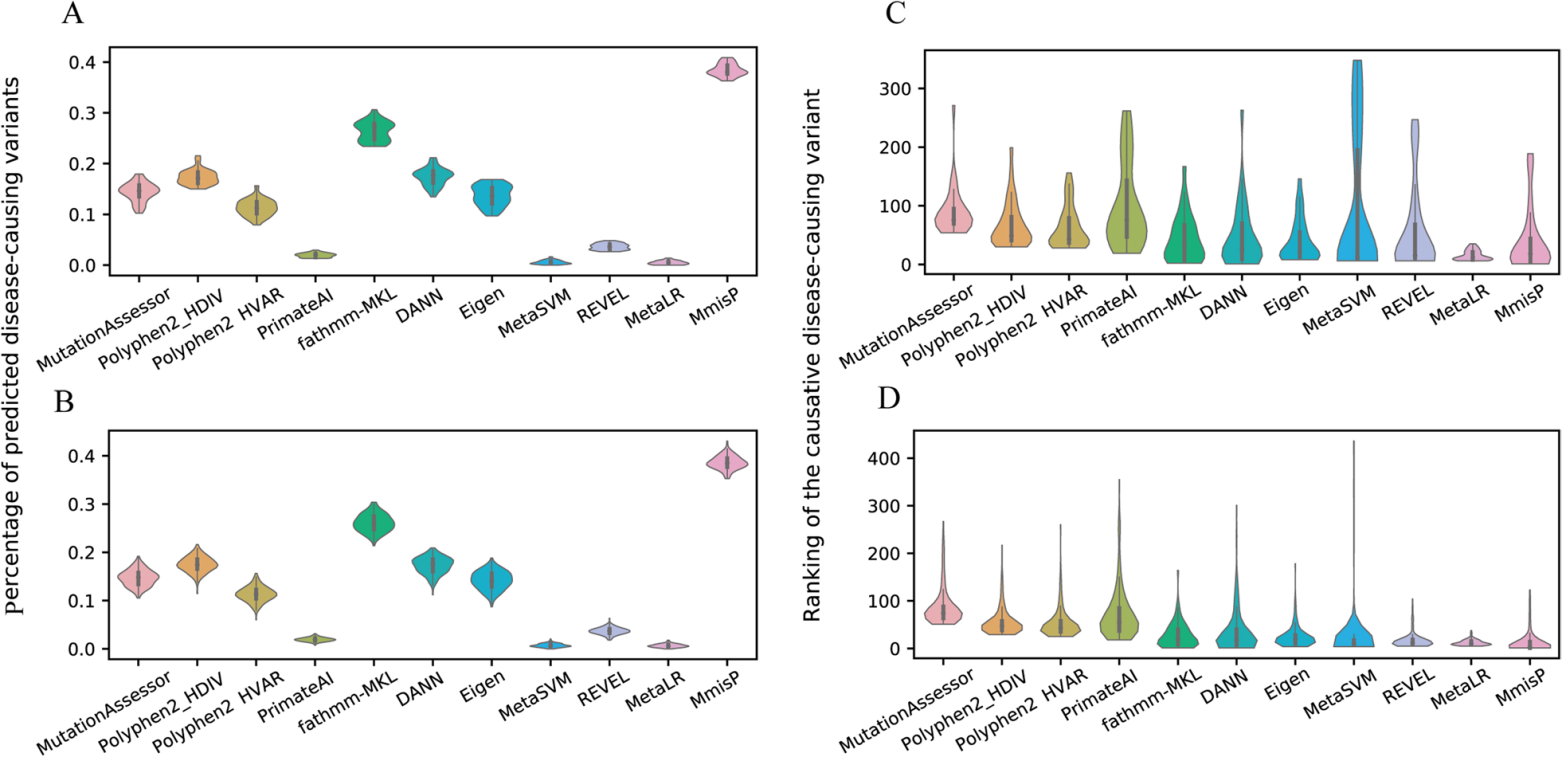
Evaluation of the different pathogenicity predictors using two simulated exomes. **A** Distribution of the percentage of predicted disease-causing variants in the Simulated_Exome29. **B** Ranking of the “causative” disease-causing variants introduced in Simulated_Exome29. **C** Distribution of the percentage of predicted disease-causing variants in the Simulated_Exome170. **D** Ranking of the “causative” disease-causing variants introduced in Simulated_Exome170

This study aimed to evaluate the performance of different pathogenicity predictors in identifying disease-causing variants in simulated exomes. To compare the length of the list of disease-causing variants identified by different predictors, we firstly calculated the percentage of disease-causing variants predicted by each predictor using the threshold values recommended. MetaLR generated the smallest candidate variant list, predicting only 0.475 ± 0.377% of Simulated_Exome29 variants as disease-causing, while MmisP predicted 38.419 ± 1.244% (Fig. 6A and Additional file 5: Table S2). Simulated_Exome170 showed a similar trend, with MetaLR predicting 0.644 ± 0.351% of variants as disease-causing, while MmisP remained the worst performer at 38.567 ± 1.366% (Fig. 6B and Additional file 6: Table S3). Additionally, we found all tools had a similar trend in the percentage of disease-causing variants predicted in both simulated disease exomes. Next, we evaluated the ability of the pathogenicity predictors to rank the ‘causative’ disease-causing variants among the top-scoring ones. After sorting the scores for each predictor, we calculated the average rank of disease-causing variants introduced in the exome simulations (Fig. 6C and Additional file 6: Table S3). In Simulated_Exome29, MmisP performed well with an average rank of 39.655 ± 55.478 (median rank: 18), which was only slightly worse than the best-performing tool, MetaLR, with an average rank of 15.414 ± 8.604 (median rank: 12), but the difference was not significant (Mann–Whitney *p* = 0.703). In Simulated_Exome170, MmisP and MetaLR both showed excellent performance (Mann–Whitney *p* = 0.027), with average ranks of 12.429 ± 21.382 and 12.162 ± 5.880(median rank: 3.5 and 10), respectively (Fig. 6D and Additional file 6: Table S3). Overall, there were significant differences in the average rank of the ‘causative’ disease-causing variants between the two simulated exomes (Additional file 6: Table S3).

## Discussion

The development of high-throughput sequencing technologies has made it possible to study the exomes of rare Mendelian disorders. However, variant annotation has resulted in many suspect variants, making it difficult for us to determine ‘causative’ disease-causing variants through manually screening or experimentally analysis. In clinical settings, the phenotype of disease is often assessed in advance through other medical tests. However, annotating all input variants using existing tools are time consuming and computationally expensive. Although several whole genome pathogenicity predictors have been developed to identify disease-causing and benign variants, their performance varies due to different construction strategies. To address this issue, our study proposes a new strategy, defining a list of nuclear genes and transcripts for primary mitochondrial disease. This improves speed of annotation and eliminates irrelevant candidate genes. Furthermore, we demonstrate that MmisP, a pathogenicity predictor specifically designed for primary mitochondrial disease, uses a specific framework to train high-quality variants and unique features, which significantly improves the prediction accuracy for nuclear gene variants compared to other genome-wide pathogenicity predictors.

The success of MmisP can be attributed to several factors. Firstly, we applied various mainstream machine learning algorithms separately to achieve the best model. Secondly, all variants used for training were screened with disease-specific labels, greatly reducing the wrong prediction of benign variants as causative factors (i.e., whether a variant has caused any disease) in the context of a specific disease. The dataset obtained by the above steps also avoids type I circular error. Thirdly, because genome-wide tools are trained on the entire genome, some genes have different functions in all related molecular mechanisms. We only consider genes whose protein expression is located in mitochondria, thereby eliminating the influence of other unrelated genes. Fourthly, our model includes disease-specific features that accurately describe the importance of genes in mitochondrial function and may lead to a more accurate interpretations of variants in critical genes. In addition, some features included in other tools may be redundant and do not make substantial contributions to model improvement. Therefore, manually filtering the feature list is an effective solution. Additionally, the K-nearest neighbor algorithm is utilized to solve the problem of missing values caused by some features not covering all genes or variants.

It is worth noting that although MmisP has been carefully developed, there are still some limitations to be addressed. Firstly, primary mitochondrial disease is just one type of Mendelian disease, so the number of available variants is much less than that of the genome-wide variants, which poses a challenge for model training. Although efforts have been made to reduce the nonlinear model into a linear model and add constraint terms, such as L1/L2 regularization, to minimize the hypothesis space, the model may still exhibit poor generalization ability. Moreover, since the training sets of Eigen, M-CAP, and REVEL is not easily to obtain, we cannot exclude overlapping variants in the testing set. In some cases, this may lead to an overestimation of the performance of these tools. For example, the sensitivity value of MutationTaster on the Vari_TestUnbalance set is 0.99 and may not accurately predict pathogenicity. Furthermore, our study found that MmisP performs poorly in the simulated exomes sets, predicting most benign variants as disease-causing. This is because we restricted the gene range trained by the model to those associated with mitochondria, resulting in fewer benign variants for training compared to genome-wide tools. However, the positive result obtained by the model was that the 'causative' disease-causing variants ranked higher in the list, making screening work less laborious. Finally, MmisP only predicts the pathogenicity prediction for missense variants, which make it challenging to consider other types of variants simultaneously. Due to the small number of other variant types, it is difficult to form a high-quality training set. In addition, the limited available features, such as population allele frequency data and segregation data, only contains values for missense variants.

In this study, we introduce a new tool for disease-specific annotation and variant pathogenicity prediction called MmisAT. This tool is specifically designed for primary mitochondrial diseases and can be easily downloaded with required files. Our study reveals the limitations of genome-wide pathogenicity predictors and emphasizes the importance and benefits of developing customized pathogenicity predictors for accurately interpreting disease-specific pathogenic variants. 

It should be noted that MmisP provides numerical evidence for the PP3/BP4 rule as a guide to interpreting specific variants in mitochondrial disease, making it more reliable than existing genome-wide pathogenicity prediction tools. However, it cannot serve as an independent clinical decision-making tool, nor can it replace the interpretation of variants in existing ACMG/AMP guidelines. Our approach introduces specific features to enhance the generality of the model and provides a new perspective for the development of the field.

By using this strategy, researchers can focus on developing new Artificial Intelligence (AI) algorithms and improving the accuracy of training data. With the continuous accumulation of available training data, it will become feasible to develop tools for predicting the incidence of genetic diseases specific to phenotypes or even specific genes in the future. In addition, we provide precomputed pathogenicity scores (all_scores.MmisP) for all rare missense variants that may be associated with primary mitochondrial disease to accurately characterize mitochondrial dysfunction. This framework can be used to develop accurate disease-specific pathogenicity predictors and improve variant interpretation of various Mendelian diseases.

### Supplementary Information

**Additional file 1.** Supplementary methods file.

**Additional file 2.** Details of MmisP's training set.

**Additional file 3.** Details of MmisP's testing set.

**Additional file 4. Table S1:** Details of the annotations contained in MmisAT.

**Additional file 5. Table S2:** The threshold for genome-wide tools.

**Additional file 6. Table S3:** The performance of various tools in two simulated exomes..

**Additional file 7. Figure S1:** The running process of MmisAT.

**Additional file 8. Figure S2:** The relationship between MmisP's performance and the size of the training set.

## Data Availability

All data and codes used in this study are available online. The source codes and example files for MmisAT are available at https://Figureshare.com/articles/software/MmisAT/21691955. The source codes and precomputed pathogenicity scores for MmisP are available at https://Figureshare.com/articles/software/MmisP/21692696.

## References

[1] Gorman GS, Chinnery PF, DiMauro S, Hirano M, Koga Y, McFarland R, Suomalainen A, Thorburn DR, Zeviani M, Turnbull DM (2016). Mitochondrial diseases. Nat Rev Dis Primers.

[2] Holt IJ, Harding AE, Morgan-Hughes JA (1988). Deletions of muscle mitochondrial DNA in patients with mitochondrial myopathies. Nature.

[3] Kaukonen J, Juselius JK, Tiranti V, Kyttälä A, Zeviani M, Comi GP, Keränen S, Peltonen L, Suomalainen A (2000). Role of adenine nucleotide translocator 1 in mtDNA maintenance. Science.

[4] Spelbrink JN, Li F-Y, Tiranti V, Nikali K, Yuan Q-P, Tariq M, Wanrooij S, Garrido N, Comi G, Morandi L (2001). Human mitochondrial DNA deletions associated with mutations in the gene encoding Twinkle, a phage T7 gene 4-like protein localized in mitochondria. Nat Genet.

[5] Van Goethem G, Dermaut B, Löfgren A, Martin J-J, Van Broeckhoven C (2001). Mutation of POLG is associated with progressive external ophthalmoplegia characterized by mtDNA deletions. Nat Genet.

[6] Rahman J, Rahman S (2018). Mitochondrial medicine in the omics era. Lancet.

[7] Gonzalez MDM, Ramos A, Aluja MP, Santos C (2020). Sensitivity of mitochondrial DNA heteroplasmy detection using Next generation sequencing. Mitochondrion.

[8] Stenton SL, Prokisch H (2020). Genetics of mitochondrial diseases: identifying mutations to help diagnosis. EBioMedicine.

[9] Wang K, Li M, Hakonarson H (2010). ANNOVAR: functional annotation of genetic variants from high-throughput sequencing data. Nucleic Acids Res.

[10] McLaren W, Gil L, Hunt SE, Riat HS, Ritchie GR, Thormann A, Flicek P, Cunningham F (2016). The ensembl variant effect predictor. Genome Biol.

[11] Cingolani P, Platts A, Wang LL, Coon M, Nguyen T, Wang L, Land SJ, Lu X, Ruden DM (2012). A program for annotating and predicting the effects of single nucleotide polymorphisms, SnpEff: SNPs in the genome of Drosophila melanogaster strain w1118; iso-2; iso-3. Fly.

[12] McCarthy DJ, Humburg P, Kanapin A, Rivas MA, Gaulton K, Cazier JB, Donnelly P (2014). Choice of transcripts and software has a large effect on variant annotation. Genome Med.

[13] Rubino F, Piredda R, Calabrese FM, Simone D, Lang M, Calabrese C, Petruzzella V, Tommaseo-Ponzetta M, Gasparre G, Attimonelli M (2012). HmtDB, a genomic resource for mitochondrion-based human variability studies. Nucleic Acids Res.

[14] Preste R, Vitale O, Clima R, Gasparre G, Attimonelli M (2019). HmtVar: a new resource for human mitochondrial variations and pathogenicity data. Nucleic Acids Res.

[15] Adzhubei IA, Schmidt S, Peshkin L, Ramensky VE, Gerasimova A, Bork P, Kondrashov AS, Sunyaev SR (2010). A method and server for predicting damaging missense mutations. Nat Methods.

[16] Kumar P, Henikoff S, Ng PC (2009). Predicting the effects of coding non-synonymous variants on protein function using the SIFT algorithm. Nat Protoc.

[17] Kircher M, Witten DM, Jain P, O'Roak BJ, Cooper GM, Shendure J (2014). A general framework for estimating the relative pathogenicity of human genetic variants. Nat Genet.

[18] Choi Y, Sims GE, Murphy S, Miller JR, Chan AP (2012). Predicting the functional effect of amino acid substitutions and indels. PLoS ONE.

[19] Bris C, Goudenege D, Desquiret-Dumas V, Charif M, Colin E, Bonneau D, Amati-Bonneau P, Lenaers G, Reynier P, Procaccio V (2018). Bioinformatics tools and databases to assess the pathogenicity of mitochondrial DNA variants in the field of next generation sequencing. Front Genet.

[20] Calabrese C, Simone D, Diroma MA, Santorsola M, Gutta C, Gasparre G, Picardi E, Pesole G, Attimonelli M (2014). MToolBox: a highly automated pipeline for heteroplasmy annotation and prioritization analysis of human mitochondrial variants in high-throughput sequencing. Bioinformatics.

[21] Castellana S, Fusilli C, Mazzoccoli G, Biagini T, Capocefalo D, Carella M, Vescovi AL, Mazza T (2017). High-confidence assessment of functional impact of human mitochondrial non-synonymous genome variations by APOGEE. PLoS Comput Biol.

[22] Navarro AM, Cámara EM, Pesini ER: MITOCLASS.1, un predictor de patogenicidad para mutaciones no sinónimas en los polipéptidos codificados por el mtDNA humano. 2016.

[23] Elson JL, Smith PM, Vila-Sanjurjo A: Heterologous Inferential Analysis (HIA) as a Method to Understand the Role of Mitochondrial rRNA Mutations in Pathogenesis. In: Mitochondrial Medicine: Volume I, Probing Mitochondrial Function. Edited by Weissig V, Edeas M. Springer New York; 2015: 369–383. 10.1007/978-1-4939-2257-4_32 25631029

[24] Martin-Navarro A, Gaudioso-Simon A, Alvarez-Jarreta J, Montoya J, Mayordomo E, Ruiz-Pesini E (2017). Machine learning classifier for identification of damaging missense mutations exclusive to human mitochondrial DNA-encoded polypeptides. BMC Bioinf.

[25] Elson JL, Smith PM, Greaves LC, Lightowlers RN, Chrzanowska-Lightowlers ZM, Taylor RW, Vila-Sanjurjo A (2015). The presence of highly disruptive 16S rRNA mutations in clinical samples indicates a wider role for mutations of the mitochondrial ribosome in human disease. Mitochondrion.

[26] Evans P, Wu C, Lindy A, McKnight DA, Lebo M, Sarmady M, Abou Tayoun AN (2019). Genetic variant pathogenicity prediction trained using disease-specific clinical sequencing data sets. Genome Res.

[27] Zhang X, Walsh R, Whiffin N, Buchan R, Midwinter W, Wilk A, Govind R, Li N, Ahmad M, Mazzarotto F (2021). Disease-specific variant pathogenicity prediction significantly improves variant interpretation in inherited cardiac conditions. Genet Med.

[28] Majithia AR, Tsuda B, Agostini M, Gnanapradeepan K, Rice R, Peloso G, Patel KA, Zhang X, Broekema MF, Patterson N (2021). Prospective functional classification of all possible missense variants in PPARG. Nat Genet.

[29] Liu HK, Dang X, Guan LP, Tian CG, Zhang SH, Ye C, Tellier LCAM, Chen F, Yang HM, Sun HX et al. A phenotype-specific framework for identifying the eye abnormalities causative nonsynonymous-variants. bioRxiv 2020;2020.2004.2013.038059.

[30] Habegger L, Balasubramanian S, Chen DZ, Khurana E, Sboner A, Harmanci A, Rozowsky J, Clarke D, Snyder M, Gerstein M (2012). VAT: a computational framework to functionally annotate variants in personal genomes within a cloud-computing environment. Bioinformatics.

[31] Landrum MJ, Chitipiralla S, Brown GR, Chen C, Gu B, Hart J, Hoffman D, Jang W, Kaur K, Liu C (2020). ClinVar: improvements to accessing data. Nucleic Acids Res.

[32] Schaafsma GC, Vihinen M (2015). VariSNP, a benchmark database for variations from dbSNP. Hum Mutat.

[33] Ioannidis NM, Rothstein JH, Pejaver V, Middha S, McDonnell SK, Baheti S, Musolf A, Li Q, Holzinger E, Karyadi D (2016). REVEL: an ensemble method for predicting the pathogenicity of rare missense variants. Am J Hum Genet.

[34] Jagadeesh KA, Wenger AM, Berger MJ, Guturu H, Stenson PD, Cooper DN, Bernstein JA, Bejerano G (2016). M-CAP eliminates a majority of variants of uncertain significance in clinical exomes at high sensitivity. Nat Genet.

[35] Rentzsch P, Witten D, Cooper GM, Shendure J, Kircher M (2019). CADD: predicting the deleteriousness of variants throughout the human genome. Nucleic Acids Res.

[36] Ionita-Laza I, McCallum K, Xu B, Buxbaum JD (2016). A spectral approach integrating functional genomic annotations for coding and noncoding variants. Nat Genet.

[37] Sundaram L, Gao H, Padigepati SR, McRae JF, Li Y, Kosmicki JA, Fritzilas N, Hakenberg J, Dutta A, Shon J (2018). Predicting the clinical impact of human mutation with deep neural networks. Nat Genet.

[38] Quang D, Chen Y, Xie X (2015). DANN: a deep learning approach for annotating the pathogenicity of genetic variants. Bioinformatics.

[39] Reva B, Antipin Y, Sander C (2011). Predicting the functional impact of protein mutations: application to cancer genomics. Nucleic Acids Res.

[40] Schwarz JM, Rodelsperger C, Schuelke M, Seelow D (2010). MutationTaster evaluates disease-causing potential of sequence alterations. Nat Methods.

[41] Shihab HA, Rogers MF, Gough J, Mort M, Cooper DN, Day IN, Gaunt TR, Campbell C (2015). An integrative approach to predicting the functional effects of non-coding and coding sequence variation. Bioinformatics.

[42] Dong C, Wei P, Jian X, Gibbs R, Boerwinkle E, Wang K, Liu X (2015). Comparison and integration of deleteriousness prediction methods for nonsynonymous SNVs in whole exome sequencing studies. Hum Mol Genet.

[43] McCormick EM, Lott MT, Dulik MC, Shen L, Attimonelli M, Vitale O, Karaa A, Bai R, Pineda-Alvarez DE, Singh LN (2020). Specifications of the ACMG/AMP standards and guidelines for mitochondrial DNA variant interpretation. Hum Mutat.

