# Supplementary material for: MmisAT and MmisP: an efficient and accurate suite of variant analysis toolkit for primary mitochondrial diseases
Source: Hum Genomics. 2023 Nov 27;17:108. doi: 10.1186/s40246-023-00557-6 (PMC10683248; doi:10.1186/s40246-023-00557-6)
Supplement: Supplementary file 1 — Additional file 1. Supplementary methods file. [file 40246_2023_557_MOESM1_ESM.docx]

**Supplementary Methods**

**Identification of nuclear genes and MANE transcripts associated with mitochondrial**

MitoCarta 3.0 Human Inventory is a data resource containing information on human-coding genes related to mitochondrial, which provides strong evidence for supporting mitochondrial localization genes [1]. It also summarizes the evidence for coding genes supporting mitochondrial localization, distribution of corresponding proteins in 14 tissues, as well as evidence for biological pathways and subcomponents. We included the 1123 genes from this resource into our candidate set of mitochondrial-related nuclear genes.

Robert et al. applied Support Vector Machine (SVM) algorithm and a collection of mitochondrial and non-mitochondrial proteins to train their model. The researchers introduced the Integrated Mitochondrial Protein Index (IMPI) as a means of determining whether the protein encoded by a gene in the entire genome is located in the mitochondria or not [2]. We downloaded the latest version of IMPI (IMPI-2020-Q3pre.xlsx), which included 1317 nuclear genes with clear evidence of mitochondrial localization, and integrated them into the candidate set of mitochondrial-related nuclear genes. Additionally, it also includes the 321 nuclear genes identified by Rahman et al. [3], as causing primary mitochondrial disease. These genes were associated with OXPHOS dysfunction, mitochondrial ultrastructure disorders, aberrations cofactor and vitamin production, or impairments in other metabolic processes in mitochondria, such as tricarboxylic acid cycle (TCA) and pyruvate metabolism. To create a comprehensive and non-redundant gene list, these three resources were integrated, and Ensembl 98 gene IDs were used as unique identity markers. The final result is a gene list consisting of 1448 mitochondria-associated nuclear genes [4].

The Matched Annotation from NCBI and EMBL-EBI (MANE) annotation project recently defined a representative transcript for each human coding gene. To ensure completeness, the MANE transcripts for each mitochondrial-related nuclear gene were manually collected. If a gene does not have a MANE transcript, the longest Uniprot-certified transcript is selected as the representative transcript [5]. To maintain consistency, Ensembl 98 Transcript ID are used, which are coordinated with the gene naming method. This resulted in a collection of 6987 transcripts, including 1448 MANE transcripts.

**Collection of routine and mitochondrial-specific annotations**

The original annotations were obtained from various data sources, but further processing was required before use. This includes removing duplicates annotations, converting coordinate systems, and other necessary steps. Additionally, several annotations were extracted to better understand the significance of rare missense variants in primary mitochondrial disease. All annotations were then grouped by function and described in further detail below.

*Basic annotation* The basic information of variants including Chromosome number, Physical position, Reference nucleotide allele, Alternative nucleotide allele, Gene name, etc, were included to give a clearly understood of variants. To enhance user experience, ID numbers from other databases (Uniprot ID, OMIM ID, rsID, etc.) are also provided to quickly retrieve variant information from other resources. Furthermore, MmisAT can annotate additional information about variants in the ClinVar [6] database, including clinical significance, phenotype list, number submitters, and review status.

*Pathogenicity predictor score* The pathogenicity predictors are genome-wide tools that distinguish disease-causing and benign variants based on evolutionarily conserved regions and functional domains. These scores are derived from dbNSFP (version: 4.1a) and can be roughly divided into three categories based on their mechanism [7]. The first category includes *in silico* machine learning variant prioritization tools, such as fathmm [8], fitCons [9], LRT [10], MutationAssessor [11] , MutationTaster [12], PolyPhen2-HDIV [13], PolyPhen2-HVAR [13], PROVEAN [14], SIFT [15], DEOGEN2 [16], ClinPred [17], LIST-S2 [18] and VEST4 [19]. The second category includes ensemble methods that combine multiple *in silico* predictors, such as CADD [20], DANN [21], Eigen [22], fathmm-MKL [23], fathmm-XF [24], GenoCanyon [25], M-CAP [26], MetaLR [27], MetaSVM [27], REVEL [28], PrimateAI [29], MVP [30] and MutPred [31]. The third category includes constraint or conservation scores for amino acid changes from GERP [32], phastCons [33], SiPhy [34], ExAC PLI [35], GeVIR [36], LOEUF [37] and CCR [38] (Table S1).

*Allele frequency* We used MmisAT to annotate allele frequency information for variants from different exome and genome sequencing data. Large-scale sequencing project, such as 1000 Genomes Project 3 (1000Gp3), ESP6500 from the Exome Variant Server, Exome Aggregation Consortium (ExAc) and Genome Aggregation Database (gnomAD) were used for this purpose (Table S1).

*Tissue expression* The study of Anderson et al. confirmed that the pathogenic variation was related to the gene expression in brain tissue and neuron cells [39]. We obtained tissue-specific gene expression data from the GTEx expression dataset and transformed it into non-parametric specificity percentage scores described by Hu et al [40]. After filtering out non-protein coding genes, we calculated non-parametric specificity scores by dividing each gene's expression value by the sum of the squares of expression values across that gene (Euclidean norm). By ranking the non-parametric specificity scores for each tissue and dividing these rankings by the number of genes, we calculated the non-parametric specificity percentile scores. Interestingly, we observed that genes with low scores (near zero) were specifically expressed, whereas genes with high scores (near one) were ubiquitously expressed or not expressed (Table S1).

*Amino acid property* We annotated the disordered residues based on the prediction from DISOPRED3, as long stretches (>30 residues) of disordered sequences exist in up to one-third of eukaryotic proteins and have been shown to be important for transcriptional regulation and signaling [41]. Residues with different solvent accessibility exhibit different degrees of variability and conservation. Generally, buried residues are usually more conservatism. Therefore, the difference between the reachable and unreachable residue replacement modes reflects the difference in the conservatism of these two types of residues. We used the SPIDER3-Single tool together with the Blast (version: 2.5.0+) sequence alignment tool and Uniref90 as a normal reference [42] to obtain the solvent-accessible surface area corresponding to each residue. To link genomic variation information to structural protein-protein interactomes, we employed the Interactome INSIDER tool[43],which enables us to identify whether variants or disease mutations are enriched in known and predicted interaction interfaces at different resolutions. Additionally, we performed multivariate statistical analysis on nearly 500 amino acid properties to generate a small set of highly interpretable numerical patterns of amino acid variation, as described by Atchley et al [44]. These high-dimensional property data were summarized in five multidimensional property covariance patterns reflecting polarity, secondary structure, molecular weight, codon diversity and electrostatic charge. To elucidate the amino acid changes, we considered the property information of two amino acids and the asymmetric substitution matrix in AAindex2 (Table S1).

*Mitochondrial-specific annotation* Balaban et al. developed  the Integrated Mitochondrial Protein Index (IMPI), which is a collection of protein-coding genes located in mammalian mitochondria and has strong evidence for cellular mitochondrial localization [2]. The IMPI incorporates various types of evidence, but its machine learning approach can lead to overfitting of training data, making it more difficult to interpret scores compared to MitoCarta 3.0. MitoCarta3.0, which provides a highly specific mitochondrial proteome reference with interpretable scores and manual curation [1]. It includes a list of mitochondrial proteins, evidence for mitochondrial localization, protein expression across 14 mouse tissues, sub-mitochondrial localization and pathway assignment. To determine known and predicted protein interactions, we used the STRING database (version: 11.0) [44]. We downloaded the '9606.protein.links.full.v11.0.txt.gz' file, which contains interaction data for human-encoded proteins. Only interactions with a composite score greater than 0.4 were retained to ensure the accuracy of the interaction network. In addition to the 1148 nuclear genes mentioned above, there are 13 encoded proteins located in mitochondria mtDNA. These proteins together constitute an interaction network responsible for the normal mitochondrial function. We propose that proteins with a core role in this interaction network are particularly important. The disruption of the more important proteins has a greater impact on mitochondrial function. To determine which proteins have core role in the interaction network, we used eight centrality metrics calculated by NetworkX software package (version: 2.5) in Python as topological information for Protein-Protein Interaction (PPI) network (Table S1).

**Training and testing sets**

We utilized three sources of missense variants: ClinVar, VariSNP and a self-curated collection of literature resources. We downloaded the ‘variant_summary.txt’ file from ClinVar version hg19 dated November 10, 2019 and filtered it based on CLINSIG, including only unambiguous pathogenic or likely pathogenic and benign or likely benign variants with no conflicting evidence. We classified Pathogenic/Likely Pathogenic as disease-causing variants and Benign/Likely Benign into benign variants as benign. After filtering, a total of 2660 variants were obtained, with 724 being benign and 1936 being disease-causing. VariSNP is a benchmark database that includes a curated set of variants dbSNP and filtered for variants associated with disease and variants found in ClinVar, Swiss-Prot, and PhenCode, ensuring that all variants are considered non-pathogenic. Using such a curated dataset is important because it enables an accurate test of the performance of different pathogenicity prediction factors without introducing bias or confounding factors.

We restricted our focus to 321 genes responsible for primary mitochondrial disease. We processed the VariSNP missense variant dataset (which did not overlap with the existing 2660 variants) and generated 2478 benign variants. From these variants, we randomly selected 1212 variants to generate the final training set Vari_Train, which contains 3872 variants with a benign to disease-causing ratio of 1:1. It is noteworthy that only 258 genes are involved in the malignant variant in Vari_Train. This is because some of the disease-causing genes lack clinical examples of missense variants and instead have other forms of variants that affect mitochondrial function. Testing set was obtained from ClinVar (From October 2019 to October 2022), but variants without pathogenicity scores were excluded from the 11 predictors used in the study. Three testing sets were created: Vari_TestUnbalance, which contains 677 benign variants and 281 pathogenic variants. After removing the variants that don't get scores in either pathogenicity predictor, we get Vari_TestBalance, which contains 256 benign variants and 239 pathogenic variants. Vari_TestThreshold contains 294 benign variants and 277 pathogenic variants, with pathogenicity scores from REVEL and M-CAP. We obtained missense variants of four genes through literature search, and most of these variants were supported by clinical examples. Through a literature search, we obtained missense variants of four widely studied genes in mitochondrial diseases (ClinGen Mitochondrial Disease Nuclear and Mitochondrial Expert Panel Specifications to the ACMG/ AMP Variant Interpretation Guidelines Version 1_ntDNA Scope: SLC19A3, PDHA1, POLG, ETHE1) and categorized these variants into a testing set named Vari_Test4Gene [45]. The Vari_Test4Gene contains 21 benign variants and 23 pathogenic variants.

These testing sets were used to compare the performance of MmisP and other pathogenicity predictors on unknown variant data and avoid type II errors.

**Features engineering**

*Feature selection* Based on the annotation provided by MmisAT, we selected 115 annotations that had a positive impact on the classification of variants as features. These selected features were labeled in the annotation file (Table S1).

*Preprocessing and feature imputation* We utilized the scikit-learn library (version: 0.24.0) in Python to extend the values of some discrete features into Euclidian space. One-hot encoding was applied to discrete features to make distance calculations between features more reasonable. Furthermore, the KNNImputer missing value imputation function in the scikit-learn library was used to estimate missing values by identifying neighboring points (k=40) and using the complete values of neighboring observations.

*Feature normalization* Due to the potential impact of the large magnitude and wide distribution of numerical values of features on the priority of some models such as Adaboost, support vector machine, KNeighbors, and Logistic Regression, we used the Z-score method for feature normalization. This approach standardized the features and conformed the data to a normal distribution. Moreover, the parameter values after standardization can reflect the contribution of different features to variant classification, facilitating feature selection.

**Machine learning model definition and selection**

We trained six machine learning algorithms - Decision Tree, Random Forest, Logistic Regression, KNeighbors, Support Vector Machine and AdaBoost on the Vari_Train missense variants using the scikit-learn library. Disease-causing variants were assigned a value of 1, while benign variants were assigned a value of 0. To improve the generalization performance of each classification algorithm, we used an exhaustive search method to obtain the optimal hyper-parameters. Specifically, we set algorithm's random seed to a fixed value, defined the range and interval of the hyper-parameters, and performed an iterative search over combinations of parameters using the GridSearchCV function, provided the best choice after an internal 5-fold cross-validation. To prove the universality of MmisP, we calculate the accuracy and its standard deviation using external 10-fold cross-validation. The score produced by machine learning algorithm ranges from 0 to 1, representing the probability of a given missense variant was classified as disease-causing. By default, missense variants with scores >0.5 are classified as disease-causing, while those with scores <0.5 are classified as benign.

**Performance** **evaluation metrics**

We compared the performance of MmisP to 11 pathogenicity predictors, including MutationAssessor, MutationTaster, PolyPhen2-HDIV, PolyPhen2-HVAR, DANN, Eigen, fathmm-MKL, M-CAP, MetaLR, MetaSVM, and PrimateAI, to determine the priority order for rare missense variants. We used the thresholds recommended by the authors to compare the classification performance of the different pathogenicity predictors on the testing sets (Table S3). To evaluate the performance of the pathogenicity predictors, we used several statistical metrics derived from the confusion matrix. Since recall and accuracy reflect different aspects of model performance, it is more appropriate to use F1 score to comprehensively evaluate the strengths and weaknesses of the model. The Matthew Correlation Coefficient (MCC) represents the correlation between true classification and the predicted classification, and is relatively robust, especially in imbalanced datasets. An MCC value of 1 indicates perfect prediction, while 0 indicates random prediction. AUC is interpreted as the probability that the current model ranks the disease-causing variant ahead of neutral variant based on calculated score. The larger AUC value, the greater the likelihood that the current model is to rank positive samples ahead of negative samples, thereby facilitating better classification. A variant was considered a true positive (TP) only when it was correctly classified as the positive class (disease-causing), while a variant was considered a true negative (TN) only when it was classified as the negative class (benign). Conversely, false positives (FP) were benign variants misclassified as disease-causing, and false negatives (FN) were disease-causing variants misclassified benign. From these different classification statistics, we calculated 12 metrics to evaluate the performance of the pathogenicity predictors.

**Recommended gene and** **threshold in ACMG/AMP Variant Interpretation Guidelines**

In 2020, the American College of Medical Genetics and Genomics (ACMG) and American Molecular Pathology (AMP) jointly developed classification criteria and guidelines for genetic variation (nDNA) in primary mitochondrial diseases, involving four nuclear genes (SLC19A3, PDHA1, POLG, ETHE1) [45]. According to these guidelines, a type of computational evidence provides strong support in determining whether a variant is disease-causing or benign (i.e., evidence PP3 and BP4). The recommended computational evidence for this purpose is REVEL pathogenicity score, where a score above 0.75 indicates disease-causing, and those below 0.15 indicates benignity. When applying a classification threshold of 75%, rare missense variants can be classified into three categories: disease-causing, benign, and uncertain. We aim to evaluate whether our MmisP tool can serve as good computational evidence for PP3 and BP4 and effectively balance type I and type II errors. Because the thresholds of some pathogenicity predictors do not fall within the 0-1 range, we will evaluate the performance of MmisP in a different background (recommended gene and threshold). Vari_Test4Gene is used to compare the performance of widely used predictors on recommended gene. Vari_TestThreshold is used to evaluate the performance of MmisP at the recommended threshold. We also included the low threshold tool M-CAP for comparison to demonstrate the importance of threshold. Here, we used a one-sided permutation test to determine whether the observed classification performance of one tool was significantly better than another. Our null hypothesis was that both pathogenicity predictors perform equally well on each evaluation metric. The null distribution was estimated by randomly exchanging 10,000 observed values between the two tools. The observed value represents the probability that the pathogenicity predictor predicts a variant as a disease-causing variant, and the p-value was estimated as the number of times the displacement difference is greater than the observed difference.

**Simulated disease exomes**

To prepare our experimental data, we used VCFtools to remove variants with minor allele frequency greater than 0.01 from 1000 Genome Project (1000G). To extract 1092 individuals from multi-sample VCF files and remove rows that do not contain variants, we used BCFtools ([www.samtools.github.io/bcftools/](http://www.samtools.github.io/bcftools/)). Using the vcf-concat Perl script in VCFtools, we concatenated the VCF files for each chromosome into one file per individual. After annotating each individual’s exome using MmisAT, we ensured the accuracy of the experiment by removing the variants overlapping with the training set from simulated exomes. We restricted variants in the simulated exomes to only correspond to 1448 mitochondria related nuclear genes. Based on a keyword search of recent literature, we found 170 new missense variants belonging to previously known mitochondrial disease-causing genes (321 nuclear genes). In the past year, we have identified 14 new disease-causing genes and found 29 related missense variants based on these new genes. Randomly select 170 of the 1092 individuals and insert the corresponding variant into the exome of each individual, resulting in Simulated_Exome170. Simulated_Exome29 is generated using the same method. After applying the circularity filter, each simulated disease exome set contained an average of 400 missense variants.

[**References**](https://academic.oup.com/bib/advance-article-abstract/doi/10.1093/bib/bbab189/6279833?redirectedFrom=fulltext#251462946)

1. Rath S, Sharma R, Gupta R, Ast T, Chan C, Durham TJ, Goodman RP, Grabarek Z, Haas ME, Hung WHW *et al*: **MitoCarta3.0: an updated mitochondrial proteome now with sub-organelle localization and pathway annotations**. *Nucleic Acids Res* 2021, **49**(D1):D1541-D1547.

2. Balaban RS: **The mitochondrial proteome: a dynamic functional program in tissues and disease states**. *Environ Mol Mutagen* 2010, **51**(5):352-359.

3. Rahman J, Rahman S: **Mitochondrial medicine in the omics era**. *Lancet* 2018, **391**(10139):2560-2574.

4. Hunt SE, McLaren W, Gil L, Thormann A, Schuilenburg H, Sheppard D, Parton A, Armean IM, Trevanion SJ, Flicek P *et al*: **Ensembl variation resources**. *Database (Oxford)* 2018, **2018**.

5. UniProt C: **UniProt: the universal protein knowledgebase in 2021**. *Nucleic Acids Res* 2021, **49**(D1):D480-D489.

6. Landrum MJ, Chitipiralla S, Brown GR, Chen C, Gu B, Hart J, Hoffman D, Jang W, Kaur K, Liu C *et al*: **ClinVar: improvements to accessing data**. *Nucleic Acids Res* 2020, **48**(D1):D835-D844.

7. Liu X, Li C, Mou C, Dong Y, Tu Y: **dbNSFP v4: a comprehensive database of transcript-specific functional predictions and annotations for human nonsynonymous and splice-site SNVs**. *Genome Med* 2020, **12**(1):103.

8. Shihab HA, Gough J, Cooper DN, Stenson PD, Barker GL, Edwards KJ, Day IN, Gaunt TR: **Predicting the functional, molecular, and phenotypic consequences of amino acid substitutions using hidden Markov models**. *Hum Mutat* 2013, **34**(1):57-65.

9. Gulko B, Hubisz MJ, Gronau I, Siepel A: **A method for calculating probabilities of fitness consequences for point mutations across the human genome**. *Nat Genet* 2015, **47**(3):276-283.

10. Chun S, Fay JC: **Identification of deleterious mutations within three human genomes**. *Genome Res* 2009, **19**(9):1553-1561.

11. Reva B, Antipin Y, Sander C: **Predicting the functional impact of protein mutations: application to cancer genomics**. *Nucleic Acids Res* 2011, **39**(17):e118.

12. Schwarz JM, Rodelsperger C, Schuelke M, Seelow D: **MutationTaster evaluates disease-causing potential of sequence alterations**. *Nat Methods* 2010, **7**(8):575-576.

13. Adzhubei IA, Schmidt S, Peshkin L, Ramensky VE, Gerasimova A, Bork P, Kondrashov AS, Sunyaev SR: **A method and server for predicting damaging missense mutations**. *Nat Methods* 2010, **7**(4):248-249.

14. Choi Y, Sims GE, Murphy S, Miller JR, Chan AP: **Predicting the functional effect of amino acid substitutions and indels**. *PLoS One* 2012, **7**(10):e46688.

15. Kumar P, Henikoff S, Ng PC: **Predicting the effects of coding non-synonymous variants on protein function using the SIFT algorithm**. *Nat Protoc* 2009, **4**(7):1073-1081.

16. Raimondi D, Tanyalcin I, Ferte J, Gazzo A, Orlando G, Lenaerts T, Rooman M, Vranken W: **DEOGEN2: prediction and interactive visualization of single amino acid variant deleteriousness in human proteins**. *Nucleic Acids Res* 2017, **45**(W1):W201-W206.

17. Alirezaie N, Kernohan KD, Hartley T, Majewski J, Hocking TD: **ClinPred: Prediction Tool to Identify Disease-Relevant Nonsynonymous Single-Nucleotide Variants**. *Am J Hum Genet* 2018, **103**(4):474-483.

18. Malhis N, Jacobson M, Jones SJM, Gsponer J: **LIST-S2: taxonomy based sorting of deleterious missense mutations across species**. *Nucleic Acids Res* 2020, **48**(W1):W154-W161.

19. Carter H, Douville C, Stenson PD, Cooper DN, Karchin R: **Identifying Mendelian disease genes with the variant effect scoring tool**. *BMC Genomics* 2013, **14 Suppl 3**(Suppl 3):S3.

20. Rentzsch P, Witten D, Cooper GM, Shendure J, Kircher M: **CADD: predicting the deleteriousness of variants throughout the human genome**. *Nucleic Acids Res* 2019, **47**(D1):D886-D894.

21. Quang D, Chen Y, Xie X: **DANN: a deep learning approach for annotating the pathogenicity of genetic variants**. *Bioinformatics* 2015, **31**(5):761-763.

22. Ionita-Laza I, McCallum K, Xu B, Buxbaum JD: **A spectral approach integrating functional genomic annotations for coding and noncoding variants**. *Nat Genet* 2016, **48**(2):214-220.

23. Shihab HA, Rogers MF, Gough J, Mort M, Cooper DN, Day IN, Gaunt TR, Campbell C: **An integrative approach to predicting the functional effects of non-coding and coding sequence variation**. *Bioinformatics* 2015, **31**(10):1536-1543.

24. Rogers MF, Shihab HA, Mort M, Cooper DN, Gaunt TR, Campbell C: **FATHMM-XF: accurate prediction of pathogenic point mutations via extended features**. *Bioinformatics* 2018, **34**(3):511-513.

25. Lu Q, Hu Y, Sun J, Cheng Y, Cheung KH, Zhao H: **A statistical framework to predict functional non-coding regions in the human genome through integrated analysis of annotation data**. *Sci Rep* 2015, **5**:10576.

26. Jagadeesh KA, Wenger AM, Berger MJ, Guturu H, Stenson PD, Cooper DN, Bernstein JA, Bejerano G: **M-CAP eliminates a majority of variants of uncertain significance in clinical exomes at high sensitivity**. *Nat Genet* 2016, **48**(12):1581-1586.

27. Dong C, Wei P, Jian X, Gibbs R, Boerwinkle E, Wang K, Liu X: **Comparison and integration of deleteriousness prediction methods for nonsynonymous SNVs in whole exome sequencing studies**. *Hum Mol Genet* 2015, **24**(8):2125-2137.

28. Ioannidis NM, Rothstein JH, Pejaver V, Middha S, McDonnell SK, Baheti S, Musolf A, Li Q, Holzinger E, Karyadi D *et al*: **REVEL: An Ensemble Method for Predicting the Pathogenicity of Rare Missense Variants**. *Am J Hum Genet* 2016, **99**(4):877-885.

29. Sundaram L, Gao H, Padigepati SR, McRae JF, Li Y, Kosmicki JA, Fritzilas N, Hakenberg J, Dutta A, Shon J *et al*: **Predicting the clinical impact of human mutation with deep neural networks**. *Nat Genet* 2018, **50**(8):1161-1170.

30. Qi H, Zhang H, Zhao Y, Chen C, Long JJ, Chung WK, Guan Y, Shen Y: **MVP predicts the pathogenicity of missense variants by deep learning**. *Nat Commun* 2021, **12**(1):510.

31. Mort M, Sterne-Weiler T, Li B, Ball EV, Cooper DN, Radivojac P, Sanford JR, Mooney SD: **MutPred Splice: machine learning-based prediction of exonic variants that disrupt splicing**. *Genome Biol* 2014, **15**(1):R19.

32. Davydov EV, Goode DL, Sirota M, Cooper GM, Sidow A, Batzoglou S: **Identifying a high fraction of the human genome to be under selective constraint using GERP++**. *PLoS Comput Biol* 2010, **6**(12):e1001025.

33. Siepel A, Bejerano G, Pedersen JS, Hinrichs AS, Hou M, Rosenbloom K, Clawson H, Spieth J, Hillier LW, Richards S *et al*: **Evolutionarily conserved elements in vertebrate, insect, worm, and yeast genomes**. *Genome Res* 2005, **15**(8):1034-1050.

34. Garber M, Guttman M, Clamp M, Zody MC, Friedman N, Xie X: **Identifying novel constrained elements by exploiting biased substitution patterns**. *Bioinformatics* 2009, **25**(12):i54-62.

35. Lek M, Karczewski KJ, Minikel EV, Samocha KE, Banks E, Fennell T, O'Donnell-Luria AH, Ware JS, Hill AJ, Cummings BB *et al*: **Analysis of protein-coding genetic variation in 60,706 humans**. *Nature* 2016, **536**(7616):285-291.

36. Abramovs N, Brass A, Tassabehji M: **GeVIR is a continuous gene-level metric that uses variant distribution patterns to prioritize disease candidate genes**. *Nat Genet* 2020, **52**(1):35-39.

37. Karczewski KJ, Francioli LC, Tiao G, Cummings BB, Alfoldi J, Wang Q, Collins RL, Laricchia KM, Ganna A, Birnbaum DP *et al*: **The mutational constraint spectrum quantified from variation in 141,456 humans**. *Nature* 2020, **581**(7809):434-443.

38. Havrilla JM, Pedersen BS, Layer RM, Quinlan AR: **A map of constrained coding regions in the human genome**. *Nat Genet* 2019, **51**(1):88-95.

39. Anderson D, Baynam G, Blackwell JM, Lassmann T: **Personalised analytics for rare disease diagnostics**. *Nat Commun* 2019, **10**(1):5274.

40. Hu X, Kim H, Stahl E, Plenge R, Daly M, Raychaudhuri S: **Integrating Autoimmune Risk Loci with Gene-Expression Data Identifies Specific Pathogenic Immune Cell Subsets**. *The American Journal of Human Genetics* 2011, **89**(4):496-506.

41. Jones DT, Cozzetto D: **DISOPRED3: precise disordered region predictions with annotated protein-binding activity**. *Bioinformatics* 2015, **31**(6):857-863.

42. Kotowski K, Smolarczyk T, Roterman-Konieczna I, Stapor K: **ProteinUnet-An efficient alternative to SPIDER3-single for sequence-based prediction of protein secondary structures**. *J Comput Chem* 2021, **42**(1):50-59.

43. Meyer MJ, Beltran JF, Liang S, Fragoza R, Rumack A, Liang J, Wei X, Yu H: **Interactome INSIDER: a structural interactome browser for genomic studies**. *Nat Methods* 2018, **15**(2):107-114.

44. Szklarczyk D, Gable AL, Nastou KC, Lyon D, Kirsch R, Pyysalo S, Doncheva NT, Legeay M, Fang T, Bork P *et al*: **The STRING database in 2021: customizable protein-protein networks, and functional characterization of user-uploaded gene/measurement sets**. *Nucleic Acids Res* 2021, **49**(D1):D605-D612.

45. McCormick EM, Lott MT, Dulik MC, Shen L, Attimonelli M, Vitale O, Karaa A, Bai R, Pineda-Alvarez DE, Singh LN *et al*: **Specifications of the ACMG/AMP standards and guidelines for mitochondrial DNA variant interpretation**. *Hum Mutat* 2020, **41**(12):2028-2057.
