# Supplementary figures and images for: MmisAT and MmisP: an efficient and accurate suite of variant analysis toolkit for primary mitochondrial diseases
Source: Hum Genomics. 2023 Nov 27;17:108. doi: 10.1186/s40246-023-00557-6 (PMC10683248; doi:10.1186/s40246-023-00557-6)

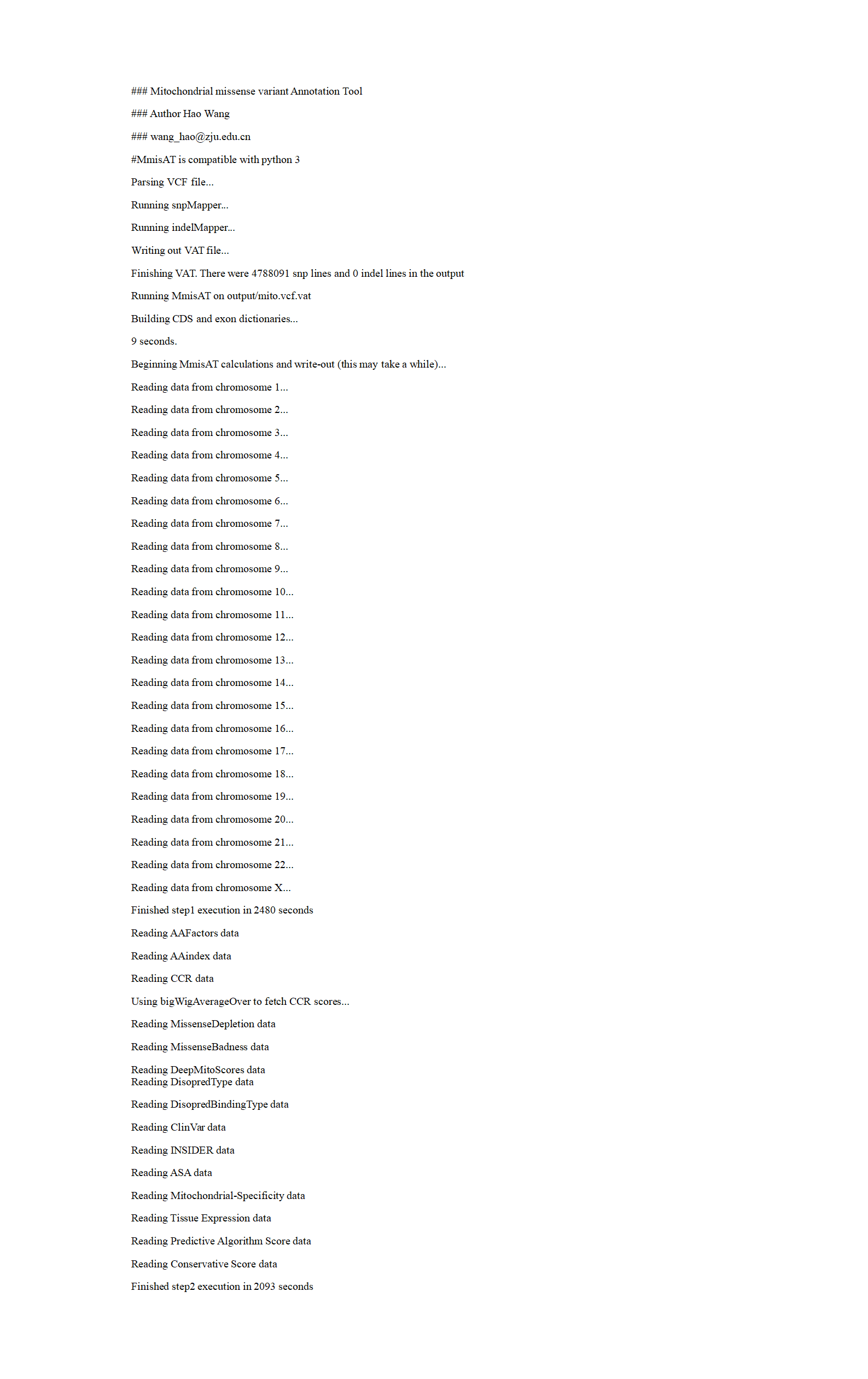

Supplement: Supplementary file 7 — Additional file 7. Figure S1: The running process of MmisAT. [file 40246_2023_557_MOESM7_ESM.tif]

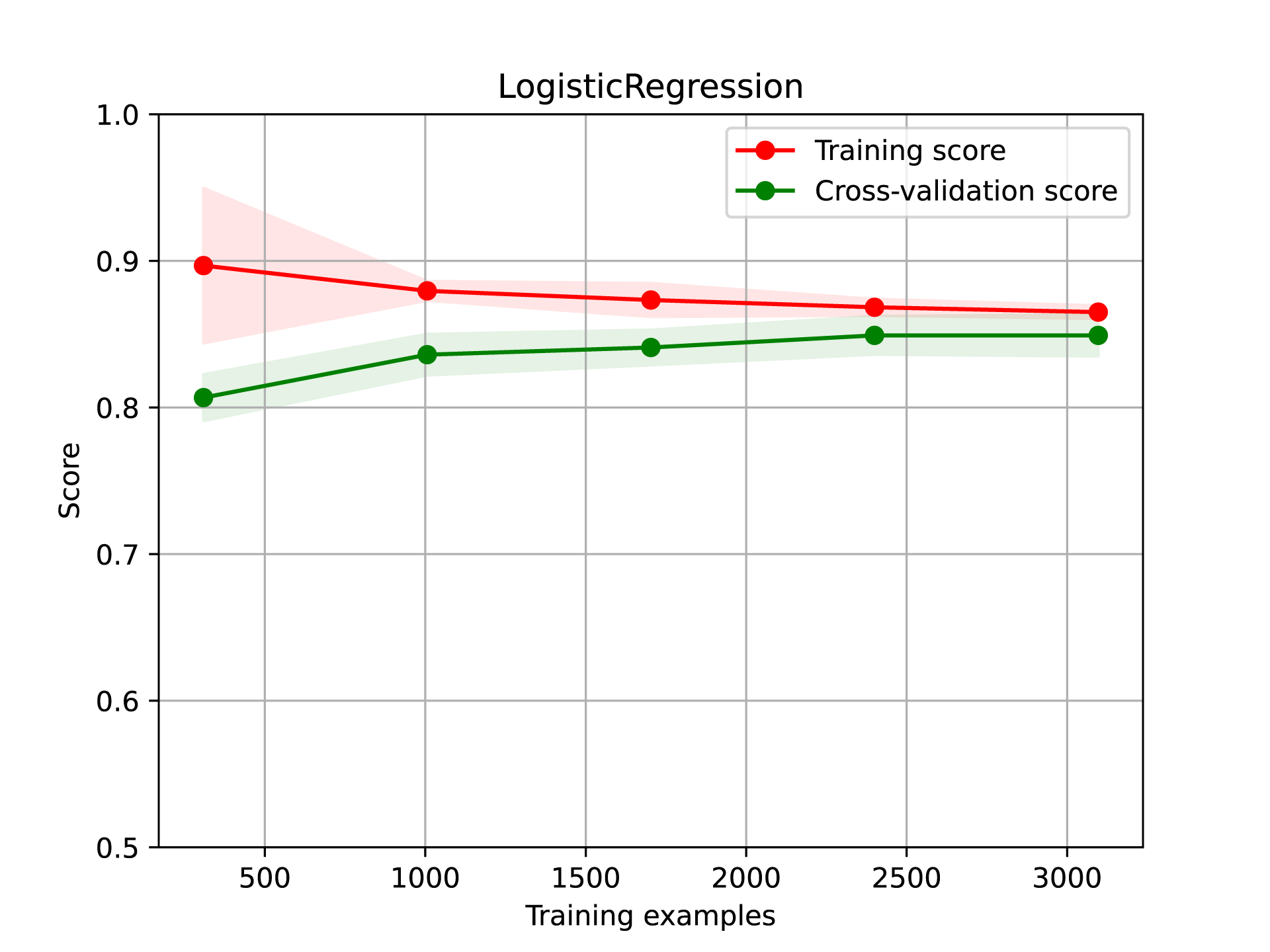

Supplement: Supplementary file 8 — Additional file 8. Figure S2: The relationship between MmisP's performance and the size of the training set. [file 40246_2023_557_MOESM8_ESM.tif]
